# Preclinical Modelling of PDA: Is Organoid the New Black?

**DOI:** 10.3390/ijms20112766

**Published:** 2019-06-05

**Authors:** Sabrina D’Agosto, Silvia Andreani, Aldo Scarpa, Vincenzo Corbo

**Affiliations:** 1 ARC-Net Research Centre, University of Verona, 37134 Verona, Italy; sabrinaluigia.dagosto@univr.it (S.D.); silvia.andreani@univr.it (S.A.); aldo.scarpa@univr.it (A.S.); 2 Department of Diagnostics and Public Health, University of Verona, 37134 Verona, Italy

**Keywords:** PDA, preclinical models, organoids, precision oncology

## Abstract

Pancreatic ductal adenocarcinoma (PDA) is a malignancy of the exocrine pancreas with the worst prognosis among all solid tumours, and soon to become the second leading cause of cancer-related deaths. A more comprehensive understanding of the molecular mechanisms underlying this disease is crucial to the development of diagnostic tools as well as to the identification of more effective therapies. High-frequency mutations in PDA occur in “undruggable” genes, and molecular subtyping based on bulk transcriptome analysis does not yet nominate valid therapeutic intervention strategies. Genome-wide sequencing studies have also demonstrated a considerable intra- and inter-patient’s genetic heterogeneity, which further complicate this dire scenario. More than in other malignancies, functionalization of the PDA genome and preclinical modelling at the individual patient level appear necessary to substantially improve survival rates for pancreatic cancer patients. Traditional human PDA models, including monolayer cell cultures and patient-derived xenografts, have certainly led to valuable biological insights in the past years. However, those model systems suffer from several limitations that have contributed to the lack of concordance between preclinical and clinical studies for PDA. Pancreatic ductal organoids have recently emerged as a reliable culture system to establish models from both normal and neoplastic pancreatic tissues. Pancreatic organoid cultures can be efficiently generated from small tissue biopsies, which opens up the possibility of longitudinal studies in individual patients. A proof-of-concept study has demonstrated that patient-derived PDA organoids are able to predict responses to conventional chemotherapy. The use of this three-dimensional culture system has already improved our understanding of PDA biology and promises to implement precision oncology by enabling the alignment of preclinical and clinical platforms to guide therapeutic intervention in PDA.

## 1. Introduction

Pancreatic ductal adenocarcinoma (PDA) is a fatal disease, the eighth most common cancer in women and currently the fourth leading cause of cancer death in men and women [1]. Although new treatment modalities have improved patients’ survival [2], PDA maintains an average 5-year survival rate of 7–8%, which is far below average survival for most common solid tumours [3]. PDA’s dismal outcome is contributed to by various factors, including late diagnosis and poor responsiveness to available therapies. Most patients (about 50%) are diagnosed with metastatic PDA and succumb to the disease within 6 to 12 months from diagnosis [4]. Life expectancy of patients whose resected tumour shows positive margins is less than 1 year, which is no different than the survival of patients with the locally advanced, unresectable disease [5]. The current standard of care for PDA consists of conventional cytotoxic drugs [6], while no effective targeted therapies have been identified to date. Therefore, a better understanding of the mechanisms underlying this disease is crucial to the development of early diagnostic tools as well as to the identification of more effective therapies. A peculiar feature of PDA is an intense desmoplastic reaction, which is known to contribute to tumour aggressiveness and resistance to therapy [7]. In addition to an extensive extracellular matrix (ECM) deposition, the stromal compartment of PDA is mainly composed of myeloid cells (e.g., neutrophils and macrophages) and cancer-associated fibroblasts (CAFs), while cytotoxic T lymphocytes are usually excluded from the tumour core [8]. Of the stromal cell types, CAFs represent the most abundant cell population. Using spontaneous mouse models of the disease, early studies showed that depletion of either CAFs or ECM components reduces the desmoplastic reaction and, accordingly, increases drug delivery to the tumour [9,10,11]. Fibroblast depletion from the PDA tumour microenvironment also increased T cell mediated anti-tumour activity in mouse models of PDA [12], thus unravelling an immune suppressive function for CAFs. Consistent with subsequent clinical failure of strategies targeting CAFs, genetic or pharmacological depletion of myofibroblasts (α-SMA expressing fibroblasts) was found to accelerate tumour progression due to the emergence of more undifferentiated tumours. While the pro- or anti-tumour role of CAFs is still debated, increasing evidences suggest functional heterogeneity within the CAFs population, which might explain the differing effects on tumour progression and resistance to therapy observed when targeting specific CAF subpopulations. PDA has a complex genomic landscape, and is frequently associated with alterations in a core set of genes, such as activating mutations of the *KRAS* oncogene (found in >90% of PDA) and inactivation of tumour suppressor genes (*TP53*, *p16/CDKN2A*, and *SMAD4*) [13]. Although activating mutations of *KRAS* are nearly universal in PDA, the mutant protein has been proven difficult to target directly. Accordingly, past and current efforts have focused on strategies aimed at targeting KRAS downstream effectors, but those have demonstrated largely ineffective due to complex mechanisms of adaptive and de novo resistance [14,15,16,17]. Recent genome-wide sequencing studies have resulted in the identification of novel somatic mutations, although in low frequency, copy number variations, structural variations, and epigenetic alterations [13,18,19] which might be used to nominate novel therapeutic strategies. The International Cancer Genome Consortium has provided a comprehensive analysis by grouping vertical data (e.g., whole genomes, exomes and mRNA expression profiles) on a large cohort of resected PDA tissues, and defined the genomic and transcriptomic landscape of PDA. Based on mRNA features, four major PDA subtypes (ADEX, Pancreatic Progenitor, Squamous, and Immunogenic) can be identified, each associated to specific molecular pathways, as well as histology and survival [20]. Despite those efforts, few therapeutic strategies have emerged based on PDA genotypes, with homologous recombination repair deficiencies (HRD) due to germline loss-of-function *BRCA* mutations, raising major expectation for the approval of the first targeted therapy in PDA with poly(ADP-ribose) polymerase (PARP) inhibitors (e.g., POLO trial: NCT02184195, olaparib). This therapy is based on a synthetic lethal interaction between PARP inhibition and loss of BRCA function, which was originally described by two different groups in 2005 [21,22]. It should be noted, however, that preclinical investigations have clearly demonstrated that therapy targeting somatic HRD in PDA is likely to be effective only when mutations in DNA repair genes have direct functional consequences on genomic integrity, thereby urging scientists to develop surrogate assays to evaluate HRD [19]. The need for establishing genotype-to-phenotype correlation is the new challenge posed by sequencing studies, and it does not exclusively apply to PDA. Genome-sequencing studies have indeed often failed to identify a clear causative relation between genetic alterations and cancers, while creating long lists of variants of uncertain significance [23]. The lack of functional evidence for the pathogenicity of genomic alterations is a major obstacle to the successful implementation of precision medicine into the clinical practice. Even when variants are reported as pathogenic, they do not always represent specific dependencies for a given cancer and, as such, they might not be useful to guide therapeutic intervention or prognostic assessment [23]. A recent experience [24] demonstrated that genomic analysis is highly informative for some cancers with targetable mutations (e.g., BRAF and EGFR), but remains insufficient to identify effective therapeutic options for the majority of patients with advanced cancers, including PDA. Even when targetable genomic alterations are discovered, patients do not always respond to therapy [25]. Strategies to confirm therapeutic efficacy or identify additional options would be beneficial to both clinicians and patients. On those grounds, we believe that the times call for an effort to functionalize the genome of PDA to evaluate the impact of genetic variants on tumour phenotypes. Given the complex interactions between individual genes and other modifying factors, including epigenetic and co-occurring genetic alterations, multiple variables should be considered when attempting at the creation of links between molecular markers and patients’ phenotypic characteristics (i.e., prognosis and response to treatments) [24]. Therefore, preclinical modelling of individual cancers should be included in the framework of personalized medicine and, as such, is required to be cost- and time-effective. Modelling of individual PDA tumours has been historically difficult due to both limited access to suitable material and the lack of a robust methodology that enables the expansion of the neoplastic cell compartment while permitting the integration of other cellular components. This review defines the conventional model systems briefly, and then focuses on 3-dimensional (3D) organoids as a newer and more reliable model system to study pancreatic cancer.

## 2. Conventional Preclinical Models

### 2.1. Monolayer Culture Models

Monolayer cell (bi-dimensional, 2D) culture systems have been a pillar of cancer research. The first human pancreatic cell line was generated in 1963 [26], and since then additional cultures have been derived from mouse and human tumours. 2D cell lines are generally easy to culture, to propagate, to cryopreserve, and to manipulate both genetically and chemically. While the use of monolayer cell lines in the study of cancer biology has led to numerous insights, 2D cell lines suffer from several limitations. Many solid tumours, especially slow-growing ones, fail to generate monolayer cell cultures, an example being primary prostate cancers [27]. For pancreatic cancer, the efficiency of generating cell lines from a resected primary tumour is quite low [28]. Difficulties in establishing PDA 2D cell cultures from resected tumours have been ascribed to an intrinsic characteristic of PDA tissues, where fibroblast-like cells often outnumber neoplastic cells. When dissociated tumour tissues are placed in conventional culture conditions, fibroblasts often outgrowth cancer cells thus impeding expansion of neoplastic cells. Furthermore, the absence of the gradients and extracellular matrix scaffold in the monolayer is another important limitation of this cell culture system. In this condition, cell–cell contacts are difficult to model, and cancer cells lack the structural organization and functional differentiation present in vivo [29]. Moreover, since most cell lines require derivation from resected tumour specimen, and most PDA patients are ineligible for surgical resection, pancreatic cell lines can only be generated from a small subset of patients. Inconsistent expression profiles in cell lines, as compared with the patient tumours, have been reported [30,31], highlighting the potential selection of more aggressive clones during the generation of the cell culture [32]. Since derivation of 2D cultures from patients has been historically difficult for PDA, most of the available literature is based on the use of a limited number of established cell lines. Contrary to common belief, a recent work by the Getz group [33] showed that established cancer cell lines are genetically heterogeneous and quickly evolve in culture. Comprehensive molecular analyses of multiple strains of established cell lines showed extensive genetic and functional diversity, affecting interpretation of drug screening analyses. Established PDA cell lines, originally propagated as monolayer cultures, have also been used to create three-dimensional culture systems either as single-cell tumour spheroids or as multicellular tumour spheroids that include stromal cell types [34,35,36,37]. These studies evidenced how the 3D environment of cancer cells critically affects their biology mostly because of different spatial organization of cell surface receptors engaged in cell-to-cell and cell-to-matrix interactions. However, several arguments advise against the use of established cell lines as system to model drug responses in vitro, even when adapted as three-dimensional cultures. Monolayer cell cultures are adapted to grow on a flat and rigid substrate, which induces dramatic mechanical stresses not usually experienced by cells in the body, and this has direct consequences on cell differentiation and clonal composition. Furthermore, phenotypic responses to drug treatment are significantly influenced by the genomic and epigenetic background of individual cancers, which can be modelled properly only by using patient-specific models. Thus, while there are many advantages to working with cancer cell lines, they may not accurately model many aspects of the PDA biology.

### 2.2. Patient-Derived Xenograft Models

An alternative method to model pancreatic cancer consists in engraftment of human tumour fragments either subcutaneously or orthotopically into immune-deficient mice (patient-derived xenografts, PDXs). PDXs retain many of the characteristics of their corresponding primary tumours, including tissue architecture and alterations in “driver” genes [38,39]. Similar to the original tumour, PDX models recapitulate the interactions of tumour cells with their surrounding stromal cells and with the extracellular matrix; depending on the degree of immunodeficiency of the host, interactions of cancer cells with the various immune cell types cannot be modelled properly. However, species specificity issues with the regard to ligands–receptor interaction (e.g., the HGF–MET axis) might cause unforeseeable problems for the translation of results obtained in this system [40]. Although the development of PDX models has improved the quality of cancer research, their application in precision oncology is restricted for different aspects. Time is a critical factor in personalized medicine. Generation of a sizeable cohort of PDXs for drug testing might require up to 8 months, which is incompatible with the need of defining treatment regimens for patients following surgery [38]. Moreover, generation of PDXs might take longer when a small biopsy is the starting material. Genomic instability of PDXs has been initially underestimated. In a recent study, it has been demonstrated that the copy number alteration landscape of PDXs changes continuously [24], and so their continuous propagation distances them from the primary tumours from which they were derived [41]. Indeed, comparison of PDXs to derived cell lines showed that PDXs do not necessarily capture the genomic landscape of primary tumours better than cell lines, in contrast to the common point of view. PDXs as complex models could be employed for studying systemic effects of a certain disease, however, limited by the additional variants emerging over the generations. Considering the role of the tumour microenvironment in evaluating the drug response [42], the absence of immune system components in PDX models makes them inappropriate for screening and functional analysis of new immune-therapeutic drugs [38].

## 3. New Preclinical Models

### 3.1. Tumour Organoids

The term “organoid” refers to a group of cells growing in a three-dimensional (3D) structure that can be generated directly from primary tissue samples, adult stem cells, or pluripotent stem cells. Organoids are self-renewing and self-organizing structures, which preserve similar appearance and functionality as the original tissue [43,44,45]. Organoids can be maintained through serial passaging and preserve genetic stability also when derived from non-neoplastic cells [46,47]. To date, different organoid models have been established from a variety of cancer tissues, including colon [48], prostate [27,49], gastric [50], breast [51,52], pancreatic [53,54], oesophageal [55], liver [56], lung [57], kidney [58], and brain cancers [59,60]. 3D organoids are usually generated by the digestion of the original tissue into small fragments that are then embedded in an extracellular matrix. Collagen type I and Matrigel (a mixture of Collagen type IV and Laminin) are the commonly used matrices for the generation of 3D structures. Also, specific growth factors and differentiation modulators are needed, which varies from a culture system to another. The Clevers’ Laboratory has been a pioneer in the field, developing a system where epithelial cells from adult tissues, self-organize in a three-dimensional structure maintaining the identity of the original tissue. In 2013, Huch et al. [61] described a methodology to grow normal pancreatic cells from mouse tissue. Later, a collaborative effort between the Clever and Tuveson laboratories [53] described the first organoid culture from mouse and human pancreatic adenocarcinoma tissues by embedding pancreas cells in Matrigel. They used growth-factor reduced Matrigel with the addition of several growth factors (e.g., EGF, FGF10), morphogens (e.g., WNT modulators, Noggin), inhibitors (e.g., the TGFβ inhbitor A8301), and supplements (e.g., B27, Nicotinamide, N-Acetyl Cysteine) to allow propagation of mouse and human pancreatic ductal cells. Using this system, normal human pancreatic ductal cells could be propagated and cryopreserved, albeit exhibiting a limited lifespan (20–25 passages, approximately 6 months) compared to normal pancreatic mouse organoids. Interestingly, human normal ductal organoids did not show mutations in cancer genes nor evident sign of genomic instability, thus representing the first system to enable cultures of normal pancreatic cells without the need for genetic manipulation. Human tumour-derived organoids, instead, could be propagated indefinitely and were found to contain mutations in PDA “drivers” and to recapitulate major pathophysiological features of corresponding tissues when transplanted in recipient mice. Noteworthy, the authors reported that orthotopic transplantation of tumour organoids into immune-deficient mice initially generated pre-invasive lesions similar to PanIN (Pancreatic Intraepithelial Neoplasia) that could progress to invasive adenocarcinoma and metastasize, thus representing a reliable model for studying cancer progression [53]. The Skala group published another methodology for growing pancreatic tumour organoids that consents propagation of fibroblasts and tumour cells together in a mix of Matrigel and culture medium. The culture medium contains 10% foetal bovine serum (FBS), and 10 ng/mL of epidermal growth factor. Generation efficiency of this culture system has not been reported [62]. A group led by Muthuswamy also developed a methodology to generate organoids from human pancreatic adenocarcinoma. They cultured neoplastic cells as an overlay in a culture medium on top of a Matrigel bed [54]. The culture medium was supplemented with B27 supplement, ascorbic acid, insulin, hydrocortisone, fibroblast growth factor 2, all-transretinoic acid, and Y267632 (Rho Kinase inhibitor). Using this methodology, organoids could be passaged and survived cryopreservation. Interestingly, this medium does not require stimulation of Wnt signalling as opposed to the Clever’s methodology. Using this culture system, they were able to identify cytosolic localization of the transcription factor SOX9 in patients bearing mutations of TP53, which was associated with poor prognosis [54]. Finally, the Kuo group published a methodology based on an air-liquid interface that consists of collagen gel-containing transwells with direct air exposure. This condition allows 3D organoids from mouse pancreata to be grown without the need for exogenous factor supplementation [63]. The culture medium contained 20% FBS and 50 μg/mL of gentamycin. Cultures were viable for up to 50 days, but could not be passaged [63]. The same group has also described an approach to culture patient-derived organoids (PDOs) from different tumour types (including intestine, stomach and pancreas) [64]. Using a Chromium Single-Cell Immune Profiling Solution, Neal and colleagues [64] demonstrated that tumour-infiltrating lymphocytes (TILs), initially retained within PDOs cultures, recapitulate the TCR (T cell receptor) repertoire of the original tumour. Thus, this methodology enables to study endogenous immune tumour micro-environmental cells, albeit immune cells are lost over cultures propagation.

### 3.2. Tumour Organoids as Preclinical Models of PDA

**Figure 1:**
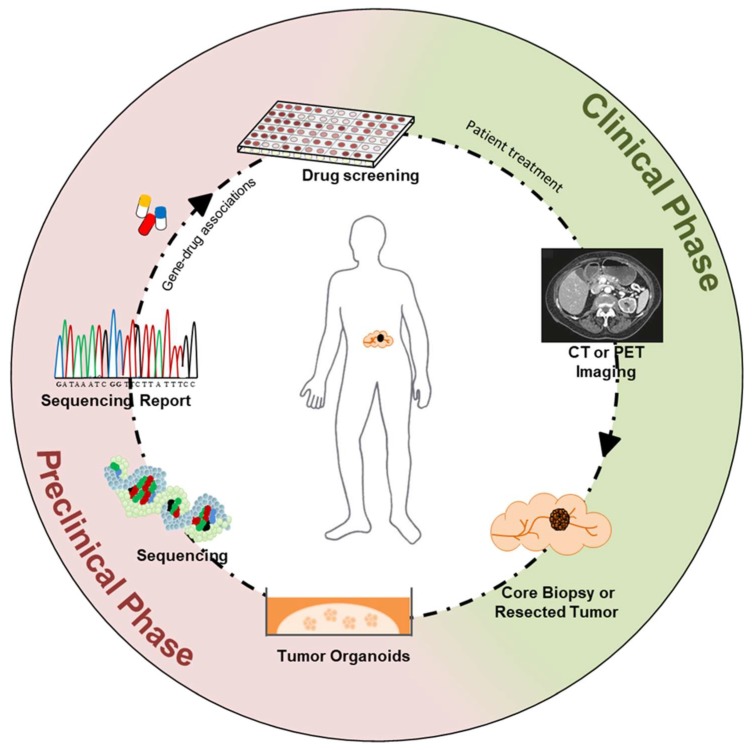
Patient-derived organoids for personalized cancer therapy. Integration of preclinical and clinical activities into a process that revolves around the patient. Patient-derived tumour organoids are rapidly established from core biopsies or resected tumour tissues. Sequencing (both DNA and RNA) is performed on organoid cultures to capture genomic and phenotypic features of the tumour, and to nominate possible therapeutic approaches. Drug-testing is performed to prioritize therapeutic intervention for the patient. Patient response to the selected treatment is evaluated also by imaging. Clinical data obtained pre- and post-treatment flow into databases that might be useful to guide further preclinical investigations. With the possibility of establishing organoid cultures from small biopsies, longitudinal sampling of the tumour also provides the opportunity for reiteration of the process.

There are several evidences that organoid cultures recapitulate major genomic and phenotypic features of patients’ tumours from which they are derived, which further supports their use in personalized cancer therapy [24,65]. In 2015, Van de Wetering and colleagues [65] generated a living organoid biobank from primary colorectal cancer patients and demonstrated that organoid cultures preserve the genomic and transcriptomic heterogeneity of the original tumour. Differently from monolayer cell cultures [33], there are evidences that gastrointestinal organoids are genetically stable upon extensive propagation in vitro [46,47]. With the possibility of generating organoids from both healthy and tumour tissues, drug screening assays and biochemical studies might also be informative of specific dependencies and vulnerabilities of the malignant state. The possibility of introducing PDOs in the framework of personalized medicine to predict clinical drug efficacy is also supported by evidences in non-cancer related field. Cystic fibrosis (CF) is a genetic disease caused by mutations of the gene encoding for the cystic fibrosis transmembrane conductance regulator (CFTR), which results in reduced protein function [66]. CF patients suffer from pulmonary infections, malnutrition, and have a low life expectancy [67]. Thousands of CFTR mutations have been described, which lead to different clinical phenotypes and different responses to available drugs [68,69]. Genotype-based prediction of drug responses is particularly challenging for CF, and measurement of rescued CFTR function upon drug treatment using PDOs has been shown to be a suitable assay to predict in vivo responses [70]. Tumour organoids established from different cancer types have shown great potential for drug screening purposes [24,65]. A seminal study from Vlachogiannis et al. [71] demonstrated that organoids established from colorectal and gastroesophageal cancers recapitulate patients’ responses to drugs. In this study [71], PDOs were generated from cancer patients enrolled in Phase 1/2 clinical trials and drug responses (anti-EGFR monoclonal antibodies, regorafenib, and TAS-102) were evaluated both ex vivo and in PDO-derived xenografts. Cross-referencing PDOs to patients’ responses, the authors showed the high predictive value of organoids-based drug testing [71]. With regard to PDA, only two of the organoid culture systems described above [53,54] have been used for preclinical investigation of drugs efficacy. In 2015, Huang et al. [54] showed similar drug sensitivity of 3D organoids and matched primary tumours. In addition to the standard of care gemcitabine, they tested tumour organoids against drugs targeting epigenetic regulators, including inhibitor of the enzymatic component of the Polycomb Repressive complex 2 (PRC2) EZH2 [54]. 3D organoids showed differential sensitivity to EZH2 inhibition and this correlated with patient’ level of H3K27me3 expression, highlighting the utility of this system for drug screening purposes. More recently, Tiriac and colleagues have established a comprehensive pancreatic PDOs library, which included models generated form small pancreatic biopsies and metastatic tumours [72]. As also reported in a previous publication [73], PDOs can be efficiently generated (success rate > 70%) from the limited amount of material procured through fine-needle aspiration biopsies, which allows derivation of PDOs from all stages of PDA [72,73]. Moreover, PDOs can be established within two weeks from tissue procurement to be rapidly available for genomic and therapeutic interrogations [73]. Sequencing analyses on 114 PDO cultures and corresponding tumour tissues demonstrated high concordance in terms of mutational landscape. Comparison of somatic copy number alterations in patient tumours and related organoids showed that genomic alterations found in clinical samples were also represented in tumour organoids. Noteworthy, since PDOs enable the selective expansion of the neoplastic compartment, this study also evidenced the possibility of using organoids to detect genetic abnormalities otherwise missed from analysis of bulk tumour tissues containing rare neoplastic cells. PDOs could be also subtyped according to available molecular classification and, overall, they were found to recapitulate the original tumours [72]. The investigation of drug sensitivities on 66 PDA-derived PDOs using common chemotherapeutic compounds (gemcitabine, nab-paclitaxel, irinotecan, 5- fluorouracil, and oxaliplatin) and cross-referencing to patients’ responses showed that PDO has a high predictive value [72]. In particular, responses to individual agents were evaluated using dose–response curves and the corresponding area under the curves (AUC) [72]. PDOs showed large variability in the responses to each chemotherapy, and only exceptional responses (lowest 33% AUC) were considered predictive of sensitivity to a given agent [72]. This study also demonstrated that no correlation could be established between specific genetic features and patients’ response to therapy. On the contrary, mRNA-based signatures derived from pharmacological perturbation of PDOs could be successfully applied to patients in order to discriminate responders from non-responders to conventional therapies. This data suggests that PDOs can be potentially aligned with clinical platforms in the framework of personalized medicine to create a bidirectional flow of information that will hopefully improve survival of PDA in the mid-term (Figure 1). 

### 3.3. Co-Culture System of Pancreatic Cancer

It is important to keep in mind that although organoids are attractive for their potential, they have an important limitation, the lack of immune and stromal cells. Even when micro-environmental cell lineages are described within PDOs, they are invariably lost over passaging [64]. To overcome this limitation, efforts have been made to develop organoid-based co-culture systems that enable integration with non-neoplastic cell types. It has been recently demonstrated that the organoid culture system can be used to model the interaction between cancer cells and the most abundant cellular components of the tumour microenvironment, CAFs. In 2017, Ohlund et al. [74] developed an organoid-based mouse co-culture system to model interaction between cancer cells and fibroblasts and observed the establishment of a symbiotic interaction between the two cell types. Varying the experimental set-up, Ohlund and colleagues further demonstrated that, in the co-culture systems, it is possible to identify two subsets of fibroblast-like cells, namely iCAFs (inflammatory CAF) and myCAF (myofibroblastic CAF), which are present in vivo. These two CAFs populations display different levels of smooth muscle actin (α-SMA) and interleukin-6 expression as a function of proximity to the cancer cells. The iCAFs population was also proposed as the pro-tumourigenic population of CAFs, being able of supporting the growth of tumour organoid in medium-depleted conditions. Building on this, Biffi and colleagues [75] demonstrated that cancer cells dictate CAF heterogeneity by releasing specific ligands, namely interleukin-1 (IL1) and transforming growth factor beta (TGFβ). Specifically, they identified IL1 as promoter of inflammatory CAFs through induction of LIF (Leukemia inhibitor factor) expression and downstream activation of the JAK/STAT signalling, whereas TGFβ antagonizes this process by downregulating IL1R1 (Interleukin-1 receptor, type 1) expression and promoting differentiation into myofibroblasts. In a more recent study [76], a different methodological approach was used to confirm that CAFs-released LIF has a pro-tumourigenic role in PDA, and that its blockade reduces tumour progression and increases efficacy of standard chemotherapy. This suggests that the organoid platform is a reliable biological system for the identification of key mechanisms in PDA and that novel therapeutic approaches for PDA should also consider the presence of functional heterogeneity within the stromal compartment. This co-culture system gives a good opportunity to investigate the cell–cell interaction and can also accommodate other cell types, including immune cells. Recently, two different platforms have been described to allow co-culture of tumour organoids from NSCLC (non-small-cell lung cancer) [77], colorectal cancer [77], and breast cancer [78] with peripheral blood lymphocytes in order to induce and analyse tumour-specific T cell responses. 

## 4. Concluding Remarks

Tumour organoid cultures have shown great potential for personalized cancer medicine, although many questions need to be addressed. Preservation of major genetic features between patient and corresponding model is a pre-requisite for a model to be potentially useful for prediction of drug sensitivity, and so is the potential of the model for retaining the clonal composition of the original tumour. Moving forward, major efforts are expected to provide information of the long-term genetic stability of this specific culture system. Another relevant question is whether the genetic and phenotypic intratumour heterogeneity of the original tumour is also preserved in PDOs. A recent paper showed that clonal organoids derived from single cells of colorectal PDO cultures displayed different responses to commonly used drugs, with different organoids from the same cancer being resistant to the treatment [79]. These results well align with the concept that large clonal diversity is a major driver of therapeutic resistance in solid tumours. Therefore, understanding the extent to which the clonal composition of the tumour is conserved in PDOs and how this is affected by drug treatments is mandatory to finally assess the PDOs predictive values and accordingly design organoid-based longitudinal studies in PDA. Preliminary evidences from the Tuveson group shows that PDOs established longitudinally over the course treatment displayed increase chemotherapy-resistance as observed in the patient [72]. Integration of organoid cultures with fibroblast-like cell types has already led to interesting insights of PDA biology, yet more complex co-cultures are needed to comprehend the reciprocal influences among the various cellular components of the PDA ecosystem, including immune cells. Other challenges to the field relate to the need for a better standardization of culture conditions, including the composition of culture-supporting matrices. Overall, a wider scientific community is expected to join this culture system to finally assess whether it will really impact patients’ outcome in the mid-term. Generation and characterization of organoids is an expensive task, which might prevent scientists from using them. Therefore, great efforts are also expected towards a reduction of culture-associated costs. This 3D culture system has already proved useful for studying the biology of PDA and a proof-of-concept study has suggested their potential use in the framework of precision medicine. Clinical trials are ongoing to evaluate the potential use of 3D pancreatic organoids as platforms for guiding therapeutic decisions in PDA.

## Abbreviations

ADEX	Aberrantly differentiated endocrine exocrine
AUC	Area under the curve
